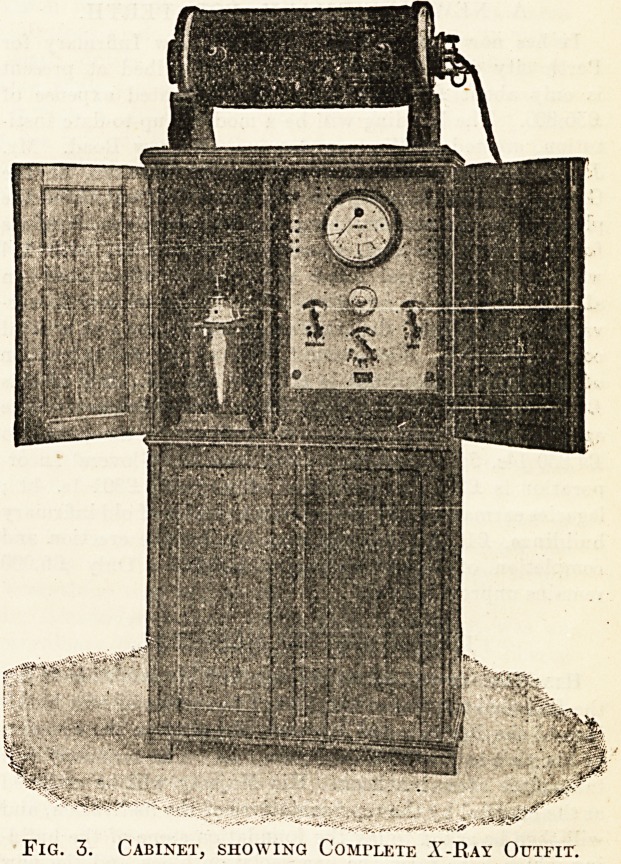# Modern X-Ray Apparatus

**Published:** 1909-09-25

**Authors:** 


### HOSPITAL ADMINISTRATION.

### CONSTRUCTION AND EQUIPMENT.

### MODERN X-RAY APPARATUS.

Very great improvements have been made in every portion of the apparatus required for the application of $x$-rays since Professor Röntgen's discovery. A large army of scientific investigators have attacked the problem, and amongst them may be mentioned Messrs. Siemens Brothers, of London and Berlin, the well-known electrical engineers. Messrs. Siemens have been connected with electricity as long as it has been a practical science. The firm have very fully equipped laboratories, and maintain a highly trained scientific staff, and amongst other improvements which they have introduced is the substitution of tantalum for the anti-cathode in place of platinum in the $x$-ray tube. As those who have used $x$-ray tubes know, the anti-cathode, the electrode which receives the cathode rays and passes them out through the glass to the object, becomes heated to a very high temperature when the tube is used for any time. The substitution of tantalum, which has a melting point 600° C. higher than that of platinum, gives tubes in which this metal is used a great advantage. In fig. 1 is shown an $x$-ray tube, as made by Messrs. Siemens, with tantalum anti-cathode, the anti-cathode having a cooling arrangement attached; the tube is also fitted with the regulating device that will now be familiar to all users of $x$-ray tubes, which enables the operator to maintain the vacuum of the tube fairly constant, or, as it is usually expressed, to keep it of uniform hardness. Another important point that those who have used $x$-rays will be familiar with is the protection of the operator and of the patient from the effects of the rays not actually required in the operation. Messrs. Siemens have given great attention to this matter also, and have devised several appliances of rubber, lead, and lead glass, which completely shield both operator and patient. Fig. 2 shows one of these, in which an $x$-ray tube is shielded by lead glass.

Fig. 1. X-Ray Tube, with Regulator, Tantalum Anti-Cathode, and Water-cooling Arrangement for the Latter.

Fig. 2. X-Ray Tube, with lead glass Shield.

Fig. 3. Cabinet, showing Complete X-Ray Outfit.

Another important point in connection with the use of $x$-rays, and particularly in the taking of radiographs, is the suppression of the inverse rays. With the induction coil, worked from an accumulator, or from a continuous current supply service, there is a small spark, when the primary current is first made, and this gives rise to what are termed inverse rays; these tend to give imperfect prints, and to render the sharp outlines that are so valuable in radiographs difficult to obtain. The trouble is overcome completely by the adoption of Dr. Albers-Schonberg's compressor, consisting of a lead-lined metal cylinder, or rectangular box, which effectually screens them off. In addition, the cylinder has an ebonite rim, by means of which the part under the rays may be compressed; the compression reduces the movement due to respiration, and thereby gives greater sharpness to the radiograph. Messrs. Siemens are the sole makers of this apparatus. It is arranged with an examining table, the compressor, with the $x$-ray tube, being moved to any part of the table, and over any part of the patient that may be required. The radiographs obtained by the aid of Dr. Albers-Schonberg's apparatus, are very clear and sharp.

The inverse rays are suppressed by other means devised by Messrs. Siemens, including an electrolytic valve, which only allows currents in one direction to pass, and they have worked out a complete series of apparatus, by means of which current may be used from any available source, and radiographs may be obtained very quickly and of sharp definition. They have also brought out cabinets, of which one is shown in fig. 3, containing a complete $x$-ray outfit. Other cabinets are arranged on trollies for use in hospital wards, and others again are made completely portable, so that the surgeon can carry them with him in his carriage.

## Figures and Tables

**Fig. 1. f1:**
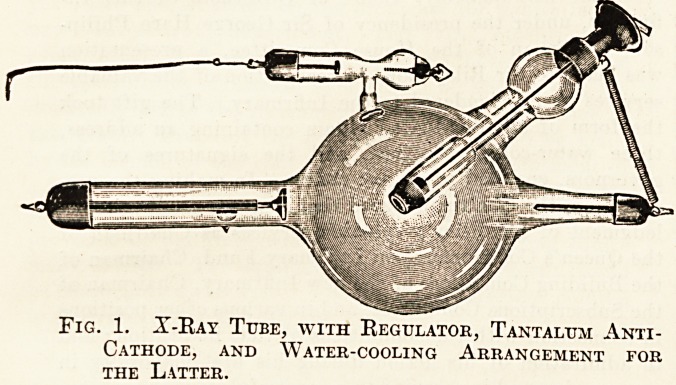


**Fig. 2. f2:**
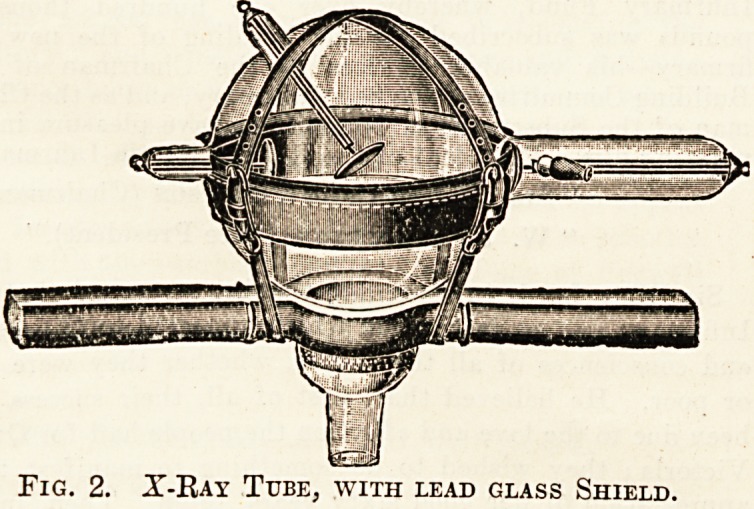


**Fig. 3. f3:**